# Diagnosis of larval migration using imaging tests

**DOI:** 10.1590/0037-8682-0326-2024

**Published:** 2025-01-27

**Authors:** Priscilla Filippo Alvim de Minas Santos, Felipe Tavares Rodrigues, Leticia Hastenreiter

**Affiliations:** 1 Universidade do Estado do Rio de Janeiro, Departamento de Dermatologia, Rio de Janeiro, RJ, Brasil.

**FIGURE 1:**
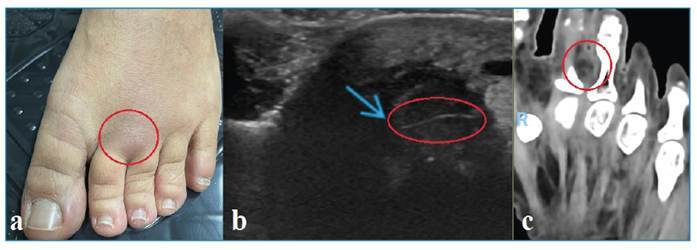
**(a)** Appearance of a foot lesion showing edema and erythema. **(b)** Demonstration of larva migrans as a hyperechoic band on ultrasound. **(c)** Demonstration of a hypointense area with hyperintense debris on magnetic resonance imaging.

A 40-year-old woman residing in a beach region had her left lower limb immobilized for 30 days after sustaining local trauma. Six months later, she developed pain, swelling, and heat on the dorsum of her left foot (Figure 1a). Ultrasonography revealed a heterogeneously hypoechoic nodular lesion, with a central echogenic linear image (7.5 × 1.0 × 0.4 mm) and hypoechoic tracts extending from the lesion to the skin of the dorsal region of the second intermediate. (Figure 1b). 

Magnetic resonance imaging revealed inflammatory edema in the subcutaneous tissue, particularly in the second intermetatarsal space, with an increase in adjacent soft tissues, and a small oval lesion corresponding to the skin maker, measuring 9.0 × 4.3 × 5.8 mm, and located at a depth of approximately 6.0 mm from the skin of the dorsal region and 27.5 mm from the skin of the plantar area (Figure 1c). Imaging aspects of the lesion on various examinations, combined with clinical data, indicated a parasitic granulomatous reaction.

Cutaneous larva migrans is a zoonotic infestation that occurs in subtropical and tropical regions and is caused by filariform hookworm larvae that penetrate and migrate into the epidermis after contact with the feces of infected animals^1^ ^,^ ^2^. The parasite is confined to the epidermis because it lacks collagenase required to break the basement membrane. Diagnosis is generally clinical, and there are few reports of complementary examinations in the literature^3^.

Herein, we report an imaging diagnosis using three complementary examinations because clinical examinations did not reveal characteristic skin lesions.
